# 208. Exposure-response relationship of ceftazidime/avibactam in adults with central nervous system infections caused by carbapenem-resistant *Klebsiella pneumoniae:* a prospective observational study

**DOI:** 10.1093/ofid/ofae631.066

**Published:** 2025-01-29

**Authors:** Nanyang Li, Lei Yang, Xu Zhu, Wanzhen Li, Xiangru Ye, Liang Gao, Lei Li, Jufang Wu, Jing Zhang, Jin Hu, Ying Mao

**Affiliations:** Huashan Hospital, Fudan University, Shanghai, Shanghai, China (People's Republic); Huashan Hospital, Fudan University, Shanghai, Shanghai, China (People's Republic); Huashan Hospital, Fudan University, Shanghai, Shanghai, China (People's Republic); Huashan Hospital, Fudan University, Shanghai, Shanghai, China (People's Republic); Huashan Hospital, Fudan University, Shanghai, Shanghai, China (People's Republic); Huashan Hospital, Fudan University, Shanghai, Shanghai, China (People's Republic); Huashan Hospital, Fudan University, Shanghai, Shanghai, China (People's Republic); Huashan Hospital, Fudan University, Shanghai, Shanghai, China (People's Republic); Huashan Hospital, Fudan University, Shanghai, Shanghai, China (People's Republic); Huashan Hospital, Fudan University, Shanghai, Shanghai, China (People's Republic); Huashan Hospital, Fudan University, Shanghai, Shanghai, China (People's Republic)

## Abstract

**Background:**

Central nervous system (CNS) infections caused by carbapenem-resistant *Klebsiella pneumoniae* (CRKP) poses significant challenges for clinicians in ICU. Achieving optimal drug exposure in the cerebrospinal fluid (CSF) is crucial for good outcomes. This study aimed to evaluate the exposure-response relationship of ceftazidime/avibactam (CZA) in plasma and CSF.

**Figure:**
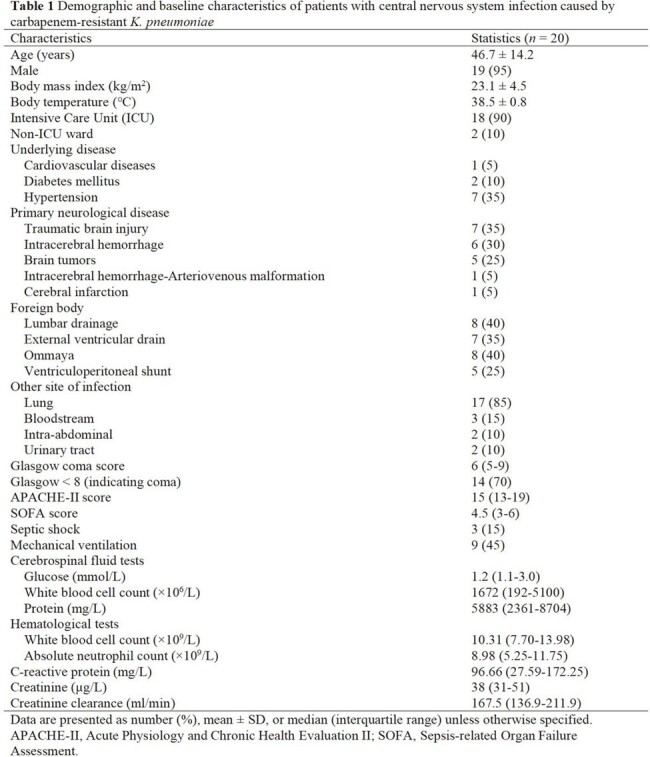

**Methods:**

All patients received CZA (2.5 g), IV infusion (over 2 hours), q8h. At steady-state after the fourth dose of CZA, the concentrations of CZA in the plasma and CSF were determined by ultra-performance liquid chromatography (UPLC) at 1, 2, 2.25, 2.5, 3, 4, 6, and 8 h post-dose. The PK/PD index of ceftazidime (CAZ) and avibactam(AVI) was calculated for each patient. Demographic, clinical efficacy, microbiological eradication, and 28-day mortality were reviewed. Blood and CSF samples were collected for calculation of PK parameters by non-compartmental pharmacokinetics (PK) modeling. The attainment of CAZ and AVI PK/PD targets in plasma and CSF was calculated to assess the exposure-response relationship of CZA and the corresponding clinical efficacy rate and bacterial eradication rate.

**Figure:**
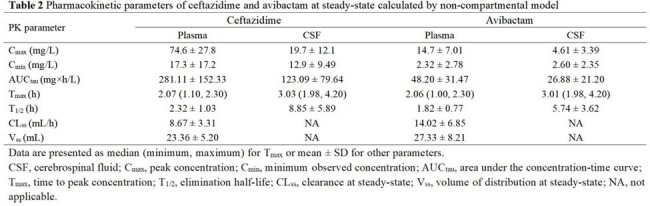

**Results:**

CAZ and AVI demonstrated good penetration into CSF, evidenced by median CSF/plasma AUC_tau_ ratio of 43.8 % and 55.8 %, respectively. CZA 2.5 g q8h achieved CAZ PK/PD target of 50%*f*T >MIC in both plasma and 50%T >MIC in CSF of all patients and reached AVI PK/PD target of 50%T >CT=1 mg/L in CSF of 80% (16/20) of patients. The dosing regimen also achieved CAZ PK/PD target of 100% *f*T >4MIC in plasma of 9 patients and 100% T >4MIC in CSF of 14 patients. At the end of the treatment, the overall clinical and microbiological efficacy rates were 80.0% and 100%, respectively. Only one case of CZA-related adverse event (*Clostridioides difficile*-associated diarrhoea) was reported.

**Figure:**
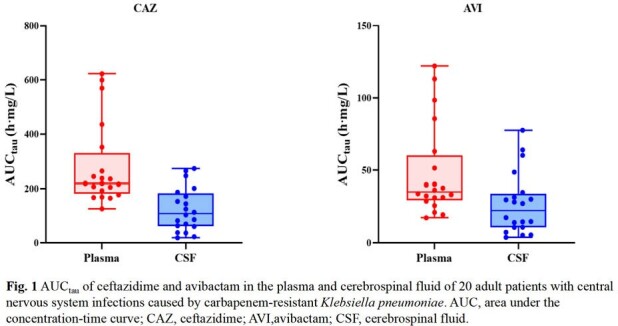

**Conclusion:**

The good CSF penetration of CZA enables the dosing regimen of CZA 2.5 g q8h a promising treatment option for CNS infections caused by CRKP.

**Figure:**
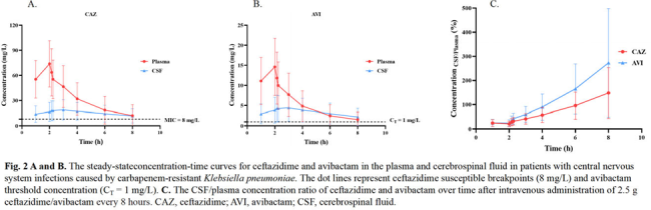

**Disclosures:**

**Nanyang Li, Master of Medicine**, TenNor Therapeutics (Suzhou) Ltd: Investigator **Jing Zhang, Doctor of Medicine**, TenNor Therapeutics (Suzhou) Ltd: Investigator

